# The Effect of Immersive Virtual Reality on Dental Anxiety and Intraoperative Pain in Adults Undergoing Local Anesthesia: A Randomized Clinical Trial

**DOI:** 10.3390/healthcare12232424

**Published:** 2024-12-03

**Authors:** Virginia Martínez-Martín, Jesús Verdejo-Herrero, Raúl Romero-del Rey, Jessica Garcia-Gonzalez, María del Mar Requena-Mullor, Raquel Alarcon-Rodriguez

**Affiliations:** 1 Department of Nursing, Physiotherapy and Medicine, Faculty of Health Sciences, University of Almeria, 04120 Almeria, Spain; vmm849@inlumine.ual.es (V.M.-M.); jvh643@inlumine.ual.es (J.V.-H.); jgg145@ual.es (J.G.-G.); mrm047@ual.es (M.d.M.R.-M.); ralarcon@ual.es (R.A.-R.); 2 Research Group CTS-1127 Epidemiology and Public Health, University of Almeria, 04120 Almeria, Spain; 3 Health Research Center (CEINSA), University of Almeria, Carretera Sacramento s/n, La Cañada, 04120 Almeria, Spain

**Keywords:** adults, anxiety, dental anxiety, pain, tooth extraction, virtual reality, oral surgery, randomized control trial

## Abstract

Aims: This study assessed the effect of immersive virtual reality (IVR) on anxiety and intraoperative pain in adult patients undergoing dental extractions with local anesthesia. Methods: In a single-blind, randomized clinical trial from September 2022 to December 2023 at a private dental clinic, 190 patients with dental anxiety were randomly assigned to either an IVR or a control group. Primary outcomes—dental anxiety and perioperative pain—were measured using the State-Trait Anxiety Inventory (STAI), Modified Dental Anxiety Scale (MDAS), and Visual Analogue Scale (VAS) before and after the procedure. Secondary outcomes included heart rate (HR), diastolic blood pressure (DBP), and systolic blood pressure (SBP), recorded at various stages. Results: The IVR group showed significant reductions in total anxiety, state anxiety, and MDAS scores compared to the control group (*p* < 0.001). Pain intensity was also lower in the IVR group (*p* = 0.03). Additionally, heart rate (HR), systolic blood pressure (SBP), and diastolic blood pressure (DBP) were consistently lower in the IVR group across different stages. Post anesthesia, the IVR group showed notably lower mean SBP and DBP values (*p* < 0.001). After surgery, the IVR group also showed lower HR (*p* = 0.01), SBP (*p* < 0.001), and DBP (*p* < 0.001) compared to the control group. Conclusions: IVR significantly reduced STAI, STAI-S, and MDAS scores and decreased intraoperative pain compared to the control group.

## 1. Introduction

Anxiety is characterized by feelings of fear and apprehension [1]. Dental anxiety (DA) is a specific emotional response to the stress of dental treatment [2] and ranks as the fifth most common cause of anxiety [3]. It can be triggered by factors such as past traumatic experiences, anxiety from family or peers, fear of the unknown, certain personality traits, a lack of understanding, coping styles, body image issues, negative media portrayals of dentists, and vulnerability in the dental chair [4,5,6,7]. Sensory stimuli, including visual cues (like needles), olfactory cues (such as the smell of eugenol), and auditory cues (like drill sounds), can also provoke anxiety [8,9,10]. A recent systematic review estimated that the global prevalence of high dental fear and anxiety is 15.3% [11].

Given that DA is one of the most common types of anxiety, identifying suitable non-invasive strategies to reduce it is both important and justified [12]. Traditionally, sedation has been used for patients who are excessively anxious or fearful of dental treatment. However, sedation can have limitations for both patients and institutions, restricting its widespread use [13]. Wong et al. [14] highlighted various strategies to reduce DA in their systematic review, including enhanced preoperative information (both content and delivery), music therapy, aromatherapy, hypnosis, auriculotherapy, acupuncture, scheduling a separate consultation day, virtual reality, oral premedication, relaxation techniques, self-efficacy improvement, and intravenous needle desensitization. Yamashita et al. [15] found that listening to music during dental extraction can decrease sympathetic activity and reduce anxiety post treatment. Similarly, Gupta et al. [16] reported that 90% of patients who listened to music during minor oral surgery expressed a desire to do so in future visits, suggesting an increase in comfort. Regarding the management of pain associated with dental extractions, various methods can be employed to alleviate discomfort. These include oral analgesics [17], topical anesthetics [18], preoperative Jidabokuippo [19], and alternative pain relief techniques [20,21,22,23]. Over the past decade, graded fear exposure, relaxation, and distraction therapies have been effectively applied using virtual reality devices, enhancing the efficiency of dental professionals [13,24,25,26,27,28,29]. These approaches have a positive impact not only on patient treatment outcomes but also on the occupational health of the professionals themselves [30].

Current research has highlighted the potential of audiovisuals, including immersive virtual reality (IVR), to decrease DA [31,32]. IVR, a technological system that offers complete sensory immersion in computer-generated virtual worlds, stands out for its ability to create immersive experiences beyond merely watching television or playing video games [28]. This technology has found increasing application in mental health treatment and clinical research [29]. Previous studies have noted that IVR distraction can modulate the processing of negative stimuli, thereby mitigating the adverse effects of intense emotions associated with dental treatment [33,34]. Furthermore, it has been suggested that the implementation of non-invasive, non-pharmacological, and inexpensive interventions, such as IVR, could improve the clinical experience of patients during dental surgery procedures, implant surgery on the first lower molar tooth [34], dental extractions [35], and maxillofacial surgery under local anesthesia [27,36]. Although the effectiveness of IVR in reducing DA in pediatric patients is well-supported by scientific evidence [37,38,39], recent systematic reviews have underscored the need for more research, especially randomized clinical trials of high methodological quality, to evaluate its effectiveness in the adult population [32,40]. Therefore, the objective of this study was to evaluate the effect of IVR on anxiety and intraoperative pain in adult patients undergoing dental extractions with local anesthesia.

## 2. Materials and Methods

### 2.1. Research Design and Ethics

A single-blind, randomized controlled clinical trial was carried out with 2 parallel groups, a group with IVR and a control group. The study protocol was approved by the Ethics and Research Committee of the University of Almería, Spain (Registration No. EFM 204). This study was registered with the Australian New Zealand Clinical Trials Registry (ACTRN12622001129774).

Participation in this study was voluntary. Participants provided written informed consent after being informed of the study’s objectives. All procedures adhered to ethical standards following the Declaration of Helsinki.

### 2.2. Participants

Recruitment for the study took place from September 2022 to December 2023. Participants had to meet the following inclusion criteria: (1) be over 18 years of age, (2) require surgical extraction [41], (3) require local anesthesia for their treatment, and (4) agree to participate and sign the informed consent form. Exclusion criteria included the following: (1) significant auditory or vision impairments, (2) coagulation problems or active infections, (3) a history of epileptic seizures, (4) undergoing oncological treatment or bisphosphonate therapy, (5) pregnancy, and (6) the inability to cooperate or comprehend the visual analogue scale (VAS) or anxiety questionnaire.

The sample size was calculated using the G*Power 3.1.9.7. program (https://www.psychologie.hhu.de/arbeitsgruppen/allgemeine-psychologie-und-arbeitspsychologie/gpower) (accessed on 15 June 2022), to estimate mean differences of 2.5 in the Spielberger State-Trait Anxiety Inventory (STAI) questionnaire score, a significance of 5% and a mean difference of 1.3 in the visual analogue scale (VAS) score, and a significance of 5% [27]. To achieve a statistical power of 80%, the largest sample size was determined for the STAI questionnaire, requiring 95 participants in each group.

### 2.3. Randomization

A statistician, with no prior contact with the participants, performed randomization using the Research Randomizer tool (www.randomizer.org) (accessed on 10 July 2022), which generated 190 codes for two groups. These codes were placed in a container. After signing informed consent, each patient drew a number from the container and was randomly assigned to either the IVR or control group.

### 2.4. Interventions

#### 2.4.1. IVR Group

Participants in this group were equipped with Shinecon VR glasses^®^ 3D, Shinecon Industrial, Fenggang (China). These glasses featured adjustable straps for a comfortable fit and could be customized for optimal focus and image quality. The device also included a vision protection system to ensure visual safety. A compatible smartphone was inserted into the front of the device, allowing participants to experience immersive 360° images of the ocean floor accompanied by relaxing sounds [27]. This immersive experience aimed to visually and auditorily isolate the patient from the external environment. The IVR intervention was administered for 20 min in the preoperative waiting room, both before and after the surgery, as well as throughout the entire dental procedure.

#### 2.4.2. Control Group

Participants in this group underwent conventional tooth extraction surgery under the same medical conditions as the IVR group but did not receive any additional intervention.

### 2.5. Data Collection

Participants were recruited from the dental service of a private clinic during their scheduled appointments for extraction surgery. All tooth extractions were performed by the same oral surgeon. Pre- and post-surgery data collection was conducted by an evaluating dentist who was not involved in the study and was unaware of the patients’ group assignments. Initially, sociodemographic variables (sex, age, and cause of extraction) and physiological responses, including heart rate (HR), systolic blood pressure (SBP), and diastolic blood pressure (DBP) were recorded. Participants also completed the State-Trait Anxiety Inventory (STAI) and the Modified Dental Anxiety Scale (MDAS).

Subsequently, the patients entered the consultation room, where a different oral surgeon performed the extraction surgery. Patients were seated in the dental chair and administered a local anesthesia injection. Once the anesthesia took effect, to maintain the blinding of the primary evaluator, a second evaluating dentist proceeded to exclusively record the physiological parameters. The oral surgeon then proceeded with the extraction surgery.

After the procedure, patients waited in the waiting room for 20 min. Following this period, they completed the STAI and MDAS questionnaires again and reported their perioperative pain sensation using the Visual Analogue Scale (VAS). Finally, the physiological parameters were recorded one last time.

The measurements of each physiological parameter were measured following the model of previous studies [16,27,42].

### 2.6. Measures

At the beginning of the study, sociodemographic variables and patient characteristics were collected, including age, sex, and causes of extraction. Physiological parameters, such as heart rate (HR), systolic blood pressure (SBP), and diastolic blood pressure (DBP), were recorded at three stages: pre-surgery, post-anesthesia, and post-surgery. HR was measured using a chest strap with a Polar H10 heart rate sensor [43], with recordings taken through the brand’s official application before surgery, after the application of anesthesia, and after surgery. SBP and DBP were measured using an Omron HBP-9031C blood pressure monitor [44]. Anxiety levels were assessed using the STAI and the MDAS. Perioperative pain was evaluated using the VAS.

#### 2.6.1. State-Trait Anxiety Inventory (STAI)

The STAI questionnaire was developed by Spielberger and colleagues in 1970 and adapted to Spanish in 1982. The Spanish version presents a Cronbach coefficient of 0.9 for the trait anxiety subscale and 0.94 for the state anxiety subscale. This self-administered questionnaire consists of 40 items, divided into two subscales of 20 items each: the state anxiety subscale (STAI-S), which evaluates the transient emotional state at a specific moment, and the trait anxiety subscale (STAI-T), which analyzes the stable propensity for anxiety. Both subscales use a 4-point Likert scale: 0 (almost never and not at all), 1 (sometimes and somewhat), 2 (often and moderately), and 3 (almost always and a lot). Total scores for each subscale vary between 0 and 60 points, with items assessing both positive and negative anxiety. Higher scores indicate higher levels of anxiety [45]. This scale has been widely used to measure anxiety in adult patients undergoing dental surgical procedures [27].

#### 2.6.2. Modified Dental Anxiety Scale (MDAS)

Additionally, the anxiety level of each patient was assessed using the Spanish version of the MDAS. This questionnaire consists of five items to evaluate the level of anxiety in different dental situations. Each question has a response on a 5-point Likert scale ranging from “not at all anxious” to “extremely anxious”. Each response is scored, and the sum of all responses is recorded. The total score on this scale ranges from 5 to 25. The reported test–retest reliability of the Spanish version of the MDAS is 0.83 and internal validity is 0.88 [46]. This scale has been widely used in patients undergoing dental surgery [47].

#### 2.6.3. Visual Analogue Scale (VAS)

Pain intensity was evaluated with the VAS after surgery and intervention. The VAS is a self-reported scale consisting of a horizontal or vertical line, usually 10 cm long (100 mm), anchored at the extremes by 2 verbal descriptors referring to the pain status. An introductory question (with or without a time recall period) asks the patient to tick the line on the point that best refers to his or her pain. The introductory question, the recall period, and the content of the external verbal descriptors vary in the literature [48]. This scale consists of a 100 mm straight line, where the patient marks with an “X” the point that best represents the intensity of pain during the procedure, with 0 indicating absence of pain and 100 the maximum pain imaginable. High test–retest reliability of the VAS scale has been reported (ICC = 0.71–0.99) [49]. This scale has been widely used in adult patients, including surgical procedures [13].

### 2.7. Data Analysis

Data analysis was carried out using the IBM SPSS 29.0 program. Descriptive analysis was calculated for the quantitative and qualitative variables, including the calculation of frequencies and percentages for the qualitative variables, and means and standard deviations (SD) for the quantitative variables. The Kolmogorov–Smirnov test assessed the normality of the continuous variables.

Baseline demographic and clinical variables were compared between groups using the Mann–Whitney U test for continuous data and the Chi-Square test (χ^2^) for categorical data. HR, DBP, SBP, STAI-S, STAI-T, STAI, MDAS, and pain were presented as means (SDs) for intragroup scores. The analyzed variables exhibited a non-normal distribution (*p* < 0.05). Intragroup comparisons were performed using the Wilcoxon test, with the effect size determined by Rosenthal’s R [50]. Comparisons between groups were calculated with the Mann–Whitney U test for non-parametric variables. A *p*-value of less than 0.05 was considered statistically significant.

## 3. Results

**Figure 1:**
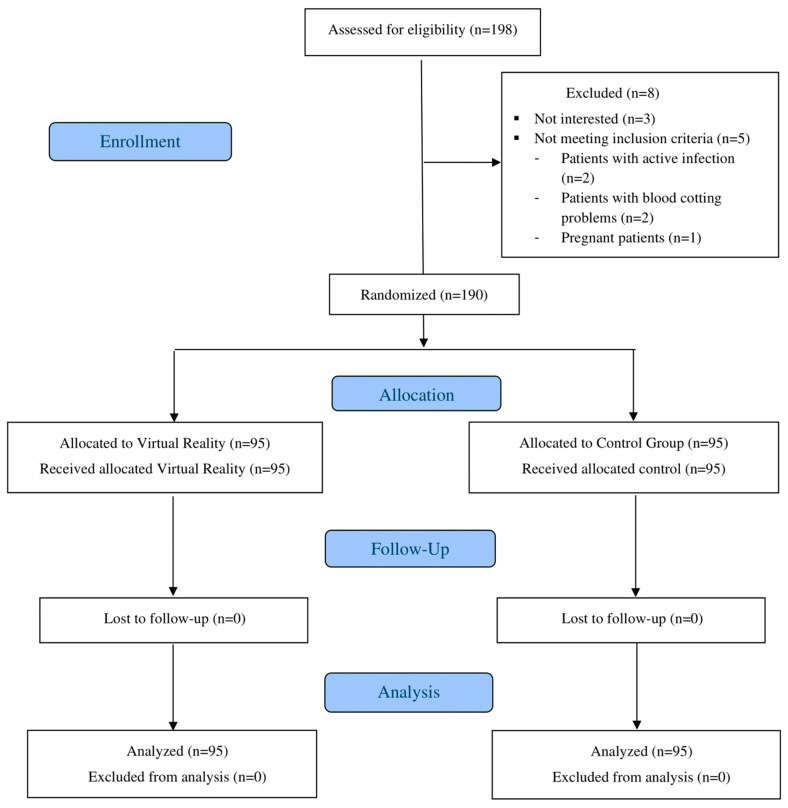
CONSORT Flow diagram.

A total of 198 patients were initially recruited for the study. However, 5 patients were withdrawn before randomization for not meeting the inclusion criteria, and 3 others declined to participate in the intervention, resulting in a final sample size of 190 participants. These 190 patients were randomly assigned to two groups, the IVR group (n = 95) and the control group (n = 95), with the latter not using an IVR device (Figure 1).

The 190 patients who participated in the study had a mean age of 48.54 (11.47) years, and 55.78% were women. The average age of the patients in the IVR group was 48.05 (11.51) years and the control group was 49.03 (11.32) years. The main cause of extraction in both groups was the presence of caries (IVR group: 57.8%, control group: 61.0%) (Table 1).

Table 2 presents the mean values of the physiological responses (HR, SBP, and DBP) in both study groups. No statistically significant differences were found between the two groups in the pre-surgery mean scores for HR, SBP, and DBP (*p* > 0.05). In the post-anesthesia measurement, SBP and DBP values were lower in the IVR group patients compared to the control group patients, with these results being statistically significant (*p* < 0.001). In the post-surgery measurement (20 min after the surgery ended), lower values were observed in all the studied physiological parameters (HR, SBP, DBP) in the IVR group patients compared to the control group, with these differences being statistically significant (*p* < 0.001).

Table 3 shows the mean values of the anxiety scales for the IVR group and the control group. When comparing anxiety levels (STAI-T, STAI-S, STAI, and MDAS) in the pre-surgery measurement, the values were similar in both the IVR group and the control group, with no statistically significant differences observed (*p* > 0.05). Post-surgery anxiety levels (STAI-S, STAI, and MDAS) were lower in the IVR group patients compared to the control group, and these results were statistically significant (STAI-S: r = 1.01; *p* < 0.01, STAI: r = 0.38; *p* = 0.005, MDAS: r = 0.39; *p* = 0.004).

Table 4 shows the comparison of mean anxiety level values between pre-surgery and post-surgery measurements in the IVR group patients and the control group patients. In the IVR group, lower post-surgery values were observed in the STAI-S, STAI, and MDAS (STAI-S: r = −0.83; *p* < 0.001, STAI: r = −0.83; *p* < 0.001, and MDAS: r = −0.78; *p* < 0.001). In the control group, anxiety values were similar between pre-surgery and post-surgery measurements, and these results were not statistically significant.

When comparing the intensity of pain during surgery between both study groups, a lower mean score was recorded in the IVR group compared to the control group (r = 0.26). This difference was statistically significant (*p* = 0.03) (Table 5).

## 4. Discussion

This study aimed to assess the effect of IVR on anxiety and intraoperative pain in adult patients undergoing dental extractions with local anesthesia, compared to a control group. After IVR intervention, mean STAI-S, STAI, and MDAS scores significantly decreased, though STAI-T scores showed no significant changes. IVR participants also reported lower intraoperative pain. Physiological measures indicated that after anesthesia, the IVR group had significantly lower SBP and DBP values than the control group, with no notable differences in HR. Post surgery, the IVR group continued to show significantly lower HR, SBP, and DBP values compared to controls.

Anxiety was measured using the STAI and MDAS scales. The IVR group showed significant reductions in both STAI-S and total STAI scores post-surgery, consistent with previous studies on IVR and third molar extractions [27]. However, no significant changes were found in STAI-T scores. This finding is consistent with a previous study [51], suggesting that STAI-T scores are less variable than STAI-S scores since STAI-T measures a personality trait reflecting relatively stable individual differences [52]. The MDAS scores also significantly decreased in the IVR group, while the control group showed no change, supporting previous research [24,25,33]. IVR provides a means to replicate, test, and modify the patient’s experience in a safe environment without compromising real-world applicability, making it an ideal tool for exposure therapies [29]. Concerning physiological responses, participants who received IVR showed significantly lower values of SBP and DBP compared to the control group after anesthesia, although no significant differences were observed in HR. After surgery, the IVR group continued to show significantly lower values of SBP and DBP compared to the control group, in addition to HR. These findings are consistent with those obtained in previous studies [27,28], who reported a decrease in blood pressure values in the IVR group during and after dental intervention.

Our results showed a significant decrease in intraoperative pain for participants who received IVR compared to the control group. This aligns with previous research that found IVR effective as a distraction during anesthesia and tooth extraction [28]. However, another study did not find improvements in pain intensity [13], possibly due to its focus on impacted third molar extractions and less dynamic IVR imagery, such as natural scenes with minimal movement. Evidence suggests that the type of IVR images may affect pain reduction [53].

Our findings have important implications for patients suffering from DA, as this anxiety is not only an emotional response to perceived threats but also has physiological and functional consequences [54]. The results of our study suggest that IVR intervention can reduce anxiety and intraoperative pain in adult patients undergoing dental surgical procedures, potentially enhancing their overall experience during these procedures under local anesthesia. IVR, noted for its ease of use and low cost, is an innovative technique that would enable dental professionals to expand their range of non-pharmacological tools, which include effective communication and music therapy [27]. Thus, our findings highlight that the implementation of IVR is not only beneficial for reducing anxiety and pain but is also a flexible strategy that can be easily incorporated into daily clinical practice. Artificial intelligence is also making a strong entrance into the field of dentistry, offering new opportunities for enhancing patient care and optimizing clinical processes [55,56]. In this regard, the combination of IVR with artificial intelligence represents an innovative approach in dentistry, with great potential to improve dental care and transform the management of clinical procedures, serving as a promising alternative that should be considered within the context of modern dental practice.

### Strengths and Limitations

The main strength of this study lies in the selection of the study population, consisting of adults with dental anxiety. There is a notable lack of studies with high methodological quality that use IVR as a distraction method in adults, compared to the abundance of research focused on the pediatric population [32,40]. Furthermore, the large sample size, designed to control alpha risk and ensure the statistical power of our results, stands out as another strength of the study. Another strength shown by the results of this study was that the IVR is a useful tool in the management of intraoperative pain, STAI-S, STAI scores, and MDAS anxiety levels in patients undergoing dental surgery. IVR could be integrated into health services to increase the quality of patient care. Future investigations could include the same experimental design for other types of treatments that require anesthesia but are generally perceived as less scary treatment, such as endodontic/restorative treatments [57], prosthetic restorations [58], or other surgical procedures [59]. However, this study is not without limitations. Due to the nature of the study, it was not possible to blind participants to the intervention received. Additionally, the control group participants did not receive any distraction or placebo techniques, which could have influenced the specific effects of IVR compared to other distraction methods. It is recommended that future studies include additional distraction techniques in the control group. Furthermore, the lack of follow-up after the intervention prevents ensuring the persistence of the results observed in terms of state anxiety in future dental visits. Future studies should aim to include follow-up assessments to evaluate the long-term persistence of reduced anxiety in subsequent dental visits. Another limitation of the study was that the analyzed physiological variables could have been influenced by factors unrelated to anxiety, such as physical movement or respiratory patterns. Future studies should include heart rate variability, which is associated with anxiety disorders [60]. Additionally, exploring the effectiveness of IVR in combination with other non-pharmacological interventions could provide a more comprehensive approach to anxiety management in dental settings. Finally, the IVR intervention, combined with devices that leverage artificial intelligence, could provide beneficial outcomes for patients undergoing dental treatments.

## 5. Conclusions

This study found that participants who received IVR during tooth extraction had significant reductions in STAI-S and STAI scores compared to the control group, along with a lower final MDAS score. Additionally, the IVR group reported reduced intraoperative pain, indicating that this intervention effectively mitigates anxiety and enhances the overall patient experience during dental procedures. These results strongly support IVR as an effective tool for managing anxiety and pain in dental settings. A clinical approach can be significantly enhanced through proactive action by anticipating and preventing health problems before they become critical (dental anxiety and intraoperative pain). By identifying risks and needs early, healthcare professionals can implement appropriate interventions, such as IVR, to help improve clinical outcomes and strengthen the patient–provider relationship. Being proactive allows for more effective and personalized health management, promoting overall wellness.

## Figures and Tables

**Table 1:** Comparison of sociodemographic data and causes of extraction among patients in the IVR and control groups.

Characteristics		IVR Group (n = 95)	Control Group (n = 95)	*p*-Value
Average age (years)		48.05 (11.51)	49.03 (11.32)	0.22 ^a^
Sex	Man	41 (43.1%)	43 (45.3%)	0.65 ^b^
	Women	54 (56.9%)	52 (54.7%)	
Cause of exodontia	Cavities	55 (57.8%)	58 (61.0%)	0.66 ^b^
	Periodontal disease	21 (22.1%)	19 (20.1%)	
	Widths	19 (20.0%)	18 (18.9%)	

The values are expressed as means (SDs); *p*-value obtained with ^a^ Mann–Whitney U or ^b^ Chi-square.

**Table 2:** Comparison of physiological responses between the IVR group and the control group.

Physiological Responses	Time Points	IVR Group	Control Group	*p*-Value
HR, beats/min.	Pre-surgery	81.76 (8.98)	81.59 (10.08)	0.45
	Post-anesthesia	80.54 (7.32)	81.65 (8.40)	0.17
	Post-surgery	78.35 (7.19)	80.88 (8.20)	0.01
SBP, mm Hg	Pre-surgery	135.74 (5.76)	135.78 (5.97)	0.48
	Post-anesthesia	128.55 (6.55)	134.88 (5.54)	<0.001
	Post-surgery	127.23 (7.55)	134.09 (6.09)	<0.001
DBP, mm Hg	Pre-surgery	78.53 (6.48)	79.45 (7.17)	0.18
	Post-anesthesia	75.16 (6.33)	79.11 (6.36)	<0.001
	Post-surgery	73.42 (5.68)	78.07 (6.64)	<0.001

The values are expressed as means (SD); *p*-value obtained with Mann–Whitney U. HR, heart rate; SBP, systolic blood pressure; DBP, diastolic blood pressure.

**Table 3:** Mean STAI and MDAS scores before and after surgery: comparison between IVR and control groups.

Time	Scale	IVR Group	Control Group	ROSENTHAL r	*p*-Value
Pre-surgery	STAI-T	22.87 (8.07)	22.65 (7.52)	−0.01	0.45
	STAI-S	23.77 (4.55)	18.31 (3.99)	−0.20	0.08
	STAI	46.64 (11.86)	41.20 (10.74)	−0.08	0.28
	MDAS	10.85 (2.04)	9.53 (1.69)	−0.17	0.11
Post-surgery	STAI-T	22.74 (8.91)	22.54 (8.43)	−0.008	0.47
	STAI-S	22.88 (4.00)	22.49 (3.72)	1.01	<0.01
	STAI	45.62 (11.90)	45.27 (10.56)	0.38	0.005
	MDAS	10.47 (2.12)	10.34 (1.90)	0.39	0.004

*p*-value obtained with Mann–Whitney U test. MDAS, Modified Dental Anxiety Scale; IVR, immersive virtual reality; STAI, State-Trait Anxiety Inventory; STAI-S, State-Trait Anxiety Inventory state subscale; STAI-T, State-Trait Anxiety Inventory trait subscale.

**Table 4:** Mean STAI and MDAS scores before and after surgery: within-group comparison for IVR and control groups.

Cluster	Anxiety	Pre-Surgery Mean (SD)	Post-Surgery Mean (SD)	ROSENTHAL r	*p*-Value
IVR group	STAI-T	22.87 (8.07)	22.74 (8.91)	−0.13	0.22
	STAI-S	23.77 (4.55)	22.88 (4.00)	−0.83	<0.001
	STAI	46.64 (11.86)	45.62 (11.90)	−0.83	<0.001
	MDAS	10.85 (2.04)	10.47 (2.12)	−0.78	<0.001
Control group	STAI-T	22.65 (7.52)	22.54 (8.43)	−0.11	0.14
	STAI-S	18.31 (3.99)	22.49 (3.72)	−0.19	0.06
	STAI	41.20 (10.74)	45.27 (10.56)	−0.19	0.06
	MDAS	9.53 (1.69)	10.34 (1.90)	−0.18	0.06

*p*-value obtained with Wilcoxon test. IVR, immersive virtual reality; MDAS, Modified Dental Anxiety Scale; STAI, Spielberger State-Trait Anxiety Inventory; STAI-T, Spielberger State-Trait Anxiety Inventory trait subscale; STAI-S, Spielberger State Anxiety Inventory-Trait state subscale.

**Table 5:** Comparison between IVR and control groups of intraoperative pain.

Cluster	Post-Surgery Pain (VAS), mm	ROSENTHAL r	*p*-Value
IVR group	12.22 (7.58)	0.26	0.03
Control group	14.14 (6.78)		

*p*-value obtained with Mann–Whitney U test. VAS, Visual Analogue Scale.

## Data Availability

For confidentiality purposes, the data are in the possession of the author (R.R.-d.R.).
